# Autism and social disconnection in interpersonal rocking

**DOI:** 10.3389/fnint.2013.00004

**Published:** 2013-02-18

**Authors:** Kerry L. Marsh, Robert W. Isenhower, Michael J. Richardson, Molly Helt, Alyssa D. Verbalis, R. C. Schmidt, Deborah Fein

**Affiliations:** ^1^Center for the Ecological Study of Perception and Action, University of ConnecticutStorrs, CT, USA; ^2^Department of Psychology, University of ConnecticutStorrs, CT, USA; ^3^Department of Psychology, University of CincinnatiCincinnati, OH, USA; ^4^Department of Psychology, College of the Holy CrossWorchester, MA, USA

**Keywords:** ASD, movement coupling, rocking synchrony, synchrony, rocking chair

## Abstract

Individuals with autism spectrum disorders (ASDs) have significant visuomotor processing deficits, atypical motoric behavior, and often substantial problems connecting socially. We suggest that the perceptual, attentional, and adaptive timing deficiencies associated with autism might directly impact the ability to become a socially connected unit with others. Using a rocking chair paradigm previously employed with typical adults, we demonstrate that typically-developing (TD) children exhibit spontaneous social rocking with their caregivers. In contrast, children diagnosed with ASD do not demonstrate a tendency to rock in a symmetrical state with their parents. We argue that the movement of our bodies is one of the fundamental ways by which we connect with our environment and, especially, ground ourselves in social environments. Deficiencies in perceiving and responding to the rhythms of the world may have serious consequences for the ability to become adequately embedded in a social context.

## Autism and social disconnection in interpersonal rocking

A defining characteristic of autism spectrum disorder (ASD) involves impairments in connecting with others, including impaired verbal and non-verbal communication, and lack of imitation and social reciprocity (APA, 2000). Early accounts of explaining such deficits seemed to partition off such deficits from perceptuo-motoric problems that also frequently occur (i.e., unusual attention processes, poor praxis and balance, and difficulty coordinating perception with action, and one limb with another; see Bhat et al., 2011), focusing instead on cognitive or motivational accounts of the social deficits. Because many social abilities such as pretend play with others can involve complex skills (e.g., joint attention, joint action, and understanding of intentions), it has been suggested that children with ASD might have a theory of mind deficit (Baron-Cohen et al., 1985; Rogers and Pennington, 1991; Williams et al., 2004). Although embodied simulation accounts that arose from research on mirror neuron processes (Rizzolatti and Craighero, 2004; Williams et al., 2004; Oberman et al., 2005) seem to give credence to theory of mind accounts, empirical evidence has failed to corroborate the role of deficiencies in these processes in the emergence of social deficits (Carpenter et al., 2001; Sebanz et al., 2005).

An adequate theoretical grounding of ASD sociality deficits is urgent in light of the increasing numbers of children being diagnosed with ASD, and the considerable resources being employed in autism interventions. Such research might have significant implications for whether the current dominant theoretical framework for developing interventions for children with ASD should continue to focus exclusively on social, cognitive, and communication skills or whether new approaches might fruitfully be added that focus on the development of a better perceptuo-motor grounding in the social world. Since communication requires movement and timing, it may well be that motoric difficulties link in crucial ways to being socially connected with others (Gernsbacher et al., 2008). In the current study, we examine whether low-level motoric processes that occur normally during social interaction—the tendency to synchronize the incidental movements of our bodies with others—is deficient in children with ASD.

Our perspective to understanding potential synchrony deficits in children with ASD starts with the assumption that humans are grounded in an environment that includes others (e.g., Marsh, 2010; Semin and Echterhoff, 2010), and that even trivial non-goal-directed movements are foundational for allowing us to be embedded in that world, to be of the world rather than standing apart from it. Crucial to a sense of connection to one's world (non-social or social) is first the ability to be able to entrain perceptually—to be able to follow and track the world. If sensory systems operate in such a way that rhythms of the world flow unexpectedly fast or slow, that one does not have sensory systems properly attuned to detect and thus synchronize with the flow of information at the proper rate, it could be uncomfortable, frightening, frustrating, or excessively arousing, which could ultimately lead one to shut off from such excessive or unpredictable stimulation.

There is substantial evidence that sensory and visual perception (e.g., timing) processes can be disrupted in children with ASD (Grossberg and Seidman, 2006). Coordination between an individual with ASD and an environmental rhythm has been examined (Gepner et al., 1995; Gepner and Mestre, 2002a,b). Typically-developing (TD) children show spontaneous entrainment of their postural sway motions to oscillatory stimuli presented on a screen; children with ASD did not exhibit such spontaneous coupling. Adults with Asperger syndrome have also been found to show impaired performance on tapping tasks that involve timing their movements to auditory stimuli (Gowen and Miall, 2005). Additionally, general deficits in motion perception have been found in children with ASD (Gepner et al., 2005; Milne et al., 2005).

As evidence from research on postural sway suggests, perceptual responses to the world are often reflected in one's movements. However, even if perceptual and visual timing systems are intact but individuals are motorically unable to be embedded in the world, and cannot properly partake in the rhythms of the world by moving their own bodies to pace themselves to it, it would be like catching a merry-go-round when we cannot run fast enough to jump on. If our bodies do not work in the regular rhythmic and symmetrical patterns that are signatures of normal rhythmic behavior (Schmidt and Richardson, 2008), a crucial and necessary condition for social connection is missing. We have hypothesized that a minimal condition for becoming a social synergy with others—a coordinated perception-action system with another (Marsh et al., 2006)—is that one is pulled into the natural orbit of another's movement rhythms—responsive to the speed of their movement and pulled to move in ways that match them temporally.

A Gibsonian ecological theory of perception (Gibson, 1979) and a dynamical systems approach to action (Warren, 2006) both posit that action is crucial for learning properly about the world, about the flow of the world, and our relationship to that world. For instance, developing proper perceptual attunement to the visual cliff comes with having crawled sufficiently to experience the optic flow in connection with our movement. Children who develop new physical capabilities encounter new possibilities for action, or affordances, particularly social affordances (Campos et al., 2000; Karasik et al., 2012). From an ecological and dynamical perspective, a child would have increased difficulty in properly developing new skills to be embedded and situated in the world, if motoric processes were off kilter.

There is substantial evidence that motoric deficiencies are often common in children with ASD. These can include fine and gross motor coordination, postural control and balance deficiencies, as well generalized difficulties performing gestures and complex movement sequences, along with bilateral arm coordination difficulties (Henderson and Sugden, 1992; Ghaziuddin et al., 1994; Ghaziuddin and Butler, 1998; Minshew et al., 2004; Jansiewicz et al., 2006; Mostofsky et al., 2006; Isenhower et al., 2012). Severity of ASD has also been linked to deficiencies synchronizing one's gestures with one's speech (de Marchena and Eigsti, 2010). Recent narrative (Bhat et al., 2011) and meta-analytic reviews (Fournier et al., 2010) of the pervasiveness of motoric difficulties in ASD suggest that motoric coordination deficits might be considered cardinal features of ASD. If perceptuo-motor deficits are integral to the social deficits of children with ASD such as deficiencies in imitation, in joint attention, and engaging in physical cooperative or verbal communication tasks (turn-taking and reciprocity) that reflect joint action (e.g., Baron-Cohen, 1989; Williams et al., 2004; Kelley et al., 2006), what might be reasonable tasks for beginning to look at such links? Many of these social tasks can require a high level of complex coordination involving attention (e.g., gaze), gesture and other complex behaviors, as well as the production of words in cognitive demanding circumstances (e.g., verbalizing thoughts). Moreover, focusing on motoric skills in the context of overtly social tasks requires that the task be one for which the child has adequate interest. Otherwise, if motoric deficiencies occur in the course of performing such a task, one could falsely assume that because the child does not perform the correct motoric behavior, they are not *able* to do so even if social interest was sufficient (Kinsbourne and Helt, 2011).

In the current paper, we focus instead on understanding the more minimal conditions that are involved in social responsiveness, focusing not on goal-directed action and all of the challenges (e.g., adequate interest in the goals) that such tasks require, but instead on inadvertent movement patterns that occur automatically under natural social interactions. An ideal task would be one in which the motoric behavior is not constrained by whether a child has shared overt goals. One approach, for example, has been to look at inadvertent social influence (movement interference) when another person (vs. an environmental stimulus) is moving in a different plane while one rhythmically moves one's arm back and forth (Gowen et al., 2008). Intriguingly, high functioning adults with ASD showed relatively limited differences in interference patterns, relative to control adults—both groups showed the typical interference effect, enhanced when the stimuli moved in a biological style of motion, and maximally impactful if the stimulus was another person's arm moving.

Whereas Gowen et al.'s task involved overt, intentional movement in the context of some other stimulus obviously moving congruently or incongruently, in our study we examined spontaneous coordination of less overt, and more incidental movement as it occurs in a social context. Focusing on simple periodic rhythmic movements is useful not only because many important movements (solitary as well as social) involve rhythmic behavior (e.g., walking or clapping), but also because considerable past research provides insight into natural dynamics of interpersonal coordination even when such movements are incidental or irrelevant to goal state (Schmidt and Richardson, 2008). The natural tendency to display such dynamics, we suggest, might be particularly informative about an individual's foundation for being socially grounded in the environment. In the current study, we use the task of spontaneously synchronizing a rocking chair to that of an adult. We use this task for two reasons. First, rocking in a chair is a natural behavior that is familiar to both children who have ASD and those who do not. Second, unlike many other tasks that may require relatively complex motor skills, or motor skills of some particular type, steadily moving a rocking chair can be achieved equally well using a variety of different methods (e.g., by pushing off with one's feet, or by merely moving one's trunk back and forth). A rocking chair is an external prop that can simultaneously amplify and simplify movement.

Although this particular paradigm has not been previously used with children, researchers have demonstrated the usefulness of a social collaborator for improving rhythmic coordination in children. For example, children's unilateral or bilateral drumming performance can be facilitated by having an adult drum with the child (Kirschner and Tomasello, 2009; Kleinspehn-Ammerlahn et al., 2011). We hypothesize that if deficiencies in the interpersonal coordination of rhythmic incidental movements occur in ASD, it may provide a window into understanding some of the minimal underlying motoric dynamic deficiencies that might restrain a child from being solidly grounded in a social world. Moreover, research with adults importantly links such interpersonal synchrony to creation of social bonds and increased susceptibility to others' influence (e.g., Hove and Risen, 2009; Miles et al., 2009; Wiltermuth and Heath, 2009; Wiltermuth, 2012).

To examine interpersonal synchrony, in the current study an adult was asked to rock at a set rhythm and children's tendency to spontaneously rock in synchrony with the adult was assessed. The synchronization model we use here is one proposed by Haken et al. (1985; HKB model) for understanding rhythmic interlimb coordination. Its modeling of the entrainment dynamics of coupled oscillators (Kugler and Turvey, 1987; Kelso, 1995) has provided an important framework for studying rhythmic coordination in adults (cf. Turvey, 1990; Amazeen et al., 1998) and children (Fitzpatrick et al., 1996; Robertson, 2001; Lantero and Ringenbach, 2007). Moreover, the model applies to both the coordination of limb movements within individuals as well as the coupling of different individuals' movements, under circumstances involving both intentional (Schmidt et al., 1990, 1998) as well as spontaneous (Schmidt and O'Brien, 1997; Richardson et al., 2005) conditions. For example, the model has been used to explain the spontaneous rocking coordination of pairs of adults in studies purportedly about rocking chair ergonomics (Richardson et al., 2007).

In the rocking chair paradigm used with adults, participants are merely asked to focus their attention on their partner's chair while each rocks at their own individual pace. Sensors tracking participants' chair movements during brief trials (e.g., 90 s) reveal that participants spontaneously synchronize rocking in a symmetrical state called in-phase behavior. In-phase rocking means that both individuals are at their maximum point forward (or backward) in their rocking cycle *relative* to each other (i.e., they are at 0° relative phase). Spontaneous synchrony in adults is evidenced by in-phase rocking at rates above 11% of a trial, with the lower range of synchronous states (e.g., 20% of a trial) occurring during spontaneous synchrony while participants are simultaneously engaged in a filler task such as mentally rehearsing memory words or forming impressions of a picture (Demos et al., 2012). When the cover story of the experiment (e.g., “rocking chair ergonomics”) does not necessitate participants doing a simultaneous task, rates of in-phase behavior can be substantially higher (e.g., 45%, Richardson et al., 2007).

In the current study we extended the rocking chair paradigm to children by assessing rocking behavior during a natural interaction with their caregiver. We predicted that children without ASD would show significantly more in-phase rocking behavior than children with ASD.

## Method

### Overview

We individually assessed children with and without ASD in their spontaneous tendency to synchronize the movement of their rocking chairs with those of a parent. The parent read a storybook to the child, while sitting in her own rocking chair and rocking throughout to a set tempo.

### Participants

Eleven children receiving a clinical diagnosis of ASD and 19 TD children participated in the study. Seven children (3 with ASD and 4 without) did not rock in the trials, leaving a sample of 8 children with ASD and 15 TD children. Participants with ASD were recruited from the ongoing University of Connecticut Early Detection ASD study (Kleinman et al., 2008). Clinicians administered the *Autism Diagnostic Observation Scale* (ADOS; Lord et al., 1999) to determine that the child met the cutoffs for and ASD. The ADOS is a semi-structured standardized assessment of communication, social interaction, and play behaviors in which a trained evaluator induces social situations that are designed to encourage the child to initiate and respond to socially. The ADOS currently has four modules corresponding to varying expressive language levels from pre-verbal/single words to fluent speech. A licensed clinician at the University of Connecticut and a doctoral student both assessed the child's score on the ADOS. A diagnosis of ASD was given if the licensed clinician determined that the child met the necessary diagnostic criteria. TD participants were a convenience sample recruited from the local university community; none of these children showed developmental delays in any domain.

Of the TD children (chronological age: 33–98 months), eight were female, the other seven were male. Children diagnosed with ASD ranged from 46 to 103 months in chronological age; two of these participants were female; the other six were male. Some analyses involved a subset of the sample matched for intellectual age. The Mullen Scales of Early Learning (Mullen, 1995), administered to all participants, assessed intellectual development on five scales: gross motor, visual reception, fine motor, receptive language, and expressive language. Fourteen children in the ASD and TD sample who could be matched to within 6 months on the visual reception subscale of the Mullen were retained as an age-equivalent-matched subsample; see Table 1 for details on this sample.

**Table 1:** **Characteristics of subsample of children: ASD and matched TD controls**.

**ASD**				**TD**			
**Child**	**Gender**	**Chronological age (months)**	**Mullen visual reception (months)**	**Child**	**Gender**	**Chronological age (months)**	**Mullen visual reception (months)**
1	M	47	27	1	F	35	27
2	F	47	29	2	F	34	33
3	M	45	30	3	M	53	34
4	M	46	46	4	M	45	40
5	F	48	48	5	F	40	42
6	M	49	60	6	M	53	60
7	M	49	61	7	M	55	66
Mean		47.4	43.0			45.2	43.1

*Note: Matching within 6 months on visual reception subscale of Mullen. The groups did not differ significantly in chronological age or visual reception scores*, |*t*|*s* < *1*.

### Procedure

**Figure 1:**
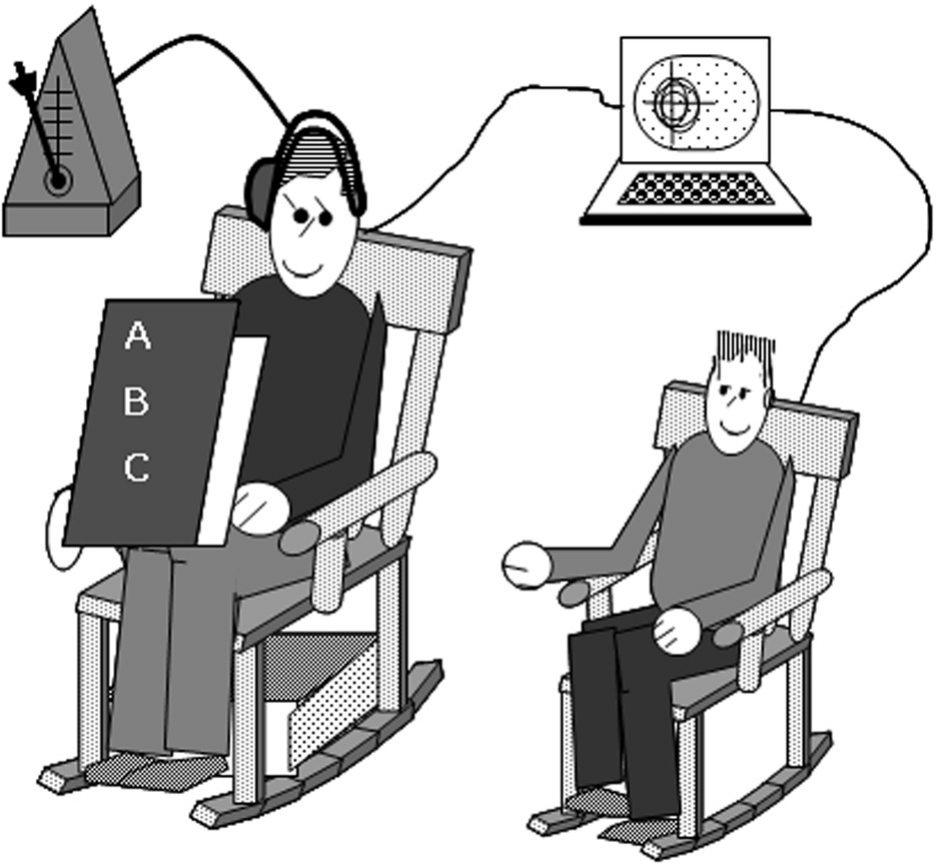
**The experimental set up.** Parents and children sat in rocking chairs. The parent read the child a story while rocking at a pace prescribed by a metronome only they can hear. Rocking movements of the parents and children were recorded via motion-tracking sensors attached unobtrusively to each chair.

A rocking chair methodology that has been used to assess spontaneous synchrony in adults (Richardson et al., 2007) was modified to examine spontaneous synchrony in children. To provide baseline rocking data, children were induced to rock continuously for 30 s in a child-sized rocking chair. For the test trials, the parent sat to the right of the child's chair, in an adult-sized rocking chair reading the child a book, and rocking at a prescribed tempo (see Figure 1). Two trials were conducted when the child's patience permitted. Each trial took between 2 and 5 min depending on the length of the book. During the trial, the parent held a children's book so that they could read it and the child could see it. Rocking chairs have a natural frequency that is determined by their construction, size, overall mass, and center of mass; the natural period of a chair's “inverted pendulum” movement can be adjusted by attaching additional weights below the center of mass. Thus, lead weights (36.3 Kg) were attached to the base of the parent's chair to allow it to rock easily at a frequency comparable to the typical rocking frequency of the child's rocking chair. In order to keep the parent rocking at a period typical of children's preferred rocking (determined to be 1.2 s in pilot testing), parents wore an earphone on one ear through which they heard a double metronome set to that period (i.e., a beep occurred every 0.6 s). Having the periods of the adult's chair move at a frequency within the range of what is natural for children allows the greatest opportunity for interpersonal synchrony to occur (Lopresti-Goodman et al., 2008). Moreover, any synchrony that occurred would be due to the child's spontaneous, unidirectional entrainment with the parent; children were not explicitly told to rock their chair during the test trials. Sensors attached to the back of each chair's headrest recorded the movement data of each rocking chair at 60 Hz (i.e., 60 samples per second) using a Polhemus Fastrak magnetic tracking system. A subsample of children also completed a bimanual drumming task alone; those data have been presented elsewhere (Isenhower et al., 2012). At the end of the session, participants received a children's book or equivalent monetary compensation for their participation.

### Coordination predictions

Predictions regarding coordinated rocking behavior were based on assumptions that if a child and adult become coordinated in their rocking, those coordination states can be understood in terms of the HKB equation for two coupled oscillators (Haken et al., 1985).

The motion equation for the HKB model (Haken et al., 1985) is as follows:

(1) $$\overset{\cdot }{\phi }=\Delta \omega -a\sin\phi -2b\sin2\phi +\sqrt{Q}\xi_{t}$$

Relative phase (ϕ) is the collective variable that captures the spatio-temporal relationship between the two component oscillators (i.e., rocking chairs in the present study). ϕ is the rate of change of relative phase. The detuning parameter, Δω, captures the difference in the natural, uncoupled, frequency of the two oscillators (Sternad et al., 1995). ξ_*t*_ is a Gaussian noise process that dictates a stochastic force of strength *Q* (Schöner et al., 1986). The relationship of the sine functions (*a* sin ϕ and 2*b* sin 2ϕ) index the relative strength of the two stable fixed point attractors of the coupled oscillators—in-phase (ϕ = 0°) and anti-phase (ϕ = 180°). At 0° relative phase, both individuals in a pair are at the same phase in their rocking cycle (e.g., both forward or backward at the same time). With rocking chair movement, spontaneous coordination is typically indicated by the amount of time that a dyad's movements are 0° relative phase. Anti-phase (being at the forward-most point in one's rocking cycle while the other is at their backward-most point) is also a stable coordination pattern that adult dyads can intentionally maintain when instructed (Richardson et al., 2007), but the HKB equation predicts that in-phase is a much stronger attractor (Haken et al., 1985).

## Results

**Figure 2:**
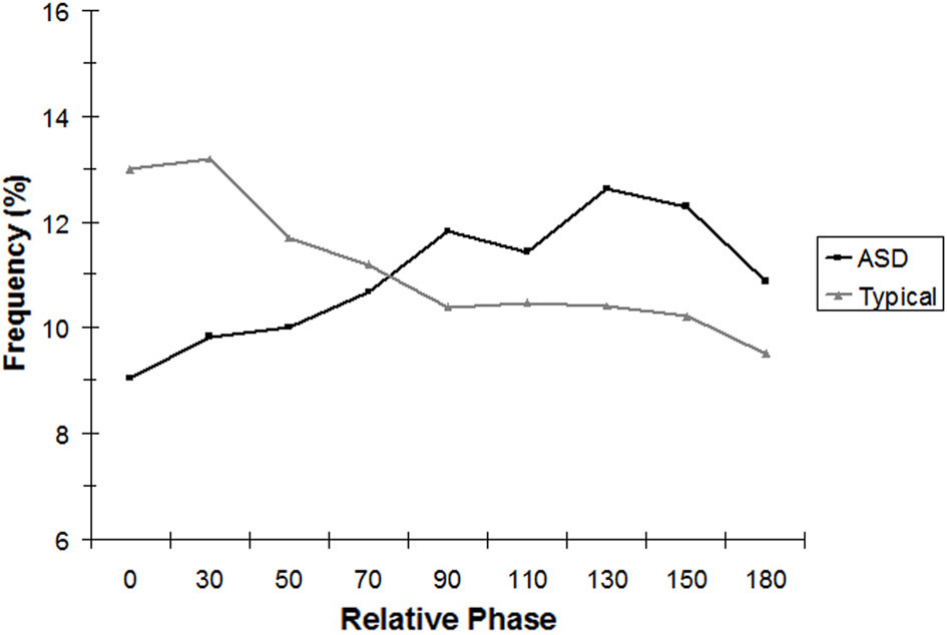
**Analysis of continuous relative phase (CRP), binned into nine equal intervals, for the complete sample**.

To test the hypothesis that TD children would show stronger in-phase coordination of their rocking chair movement with their parents than ASD children would exhibit with their parents, continuous relative phase (CRP) was analyzed on the forward/backward dimension of each dyad's movements. Children did not rock continuously throughout the trials. Children with ASD rocked an average of 42.0% (*SD* = 27.1%) of the time whereas TD children rocked an average of 47.9% of the time (*SD* = 26.8%). This difference was not significant, *t*_(21)_ = 0.51, *p* = 0.62, nor were differences (46.4% vs. 56.3%) significant in the matched sample, |*t*| < 1. Thus, comparable amounts of data were available in both groups of children to allow for analysis of bouts of continual rocking. CRP was used to calculate the average amount of time the dyad spent in a given relative phase in these bouts (with each rocking segment weighted by its relative length) using 9 bins in 20° increments arrayed from in-phase (10° either side of 0°) to anti-phase (10° either side of 180°). A 2 (Group) × 9 (Phase Region) mixed analysis of variance conducted on CRP for the full sample with phase region as a within-subjects factor revealed only a significant interaction between group and phase region, [*F*_(8, 168)_ = 5.49, *p* < 0.01]. As Figure 2 indicates, relative to children with ASD, TD children spent more time rocking in-phase with their parent. The pattern that occurred is illustrated by a significant linear contrast for the phase × group interaction, [*F*_(1, 21)_ = 9.11, *p* < 0.01]. The linear contrast tests the prediction of a continual decrease in occurrence of behavior for each relative phase region, as that region shifts further away from 0°. The linear trend of phase bin was significant for TD children only and revealed the typical pattern found for relative interpersonal coordination in adults: As relative phase values shifted away from in-phase, there was a linear decrease in the percentage of time this occurred throughout the trial.

**Figure 3:**
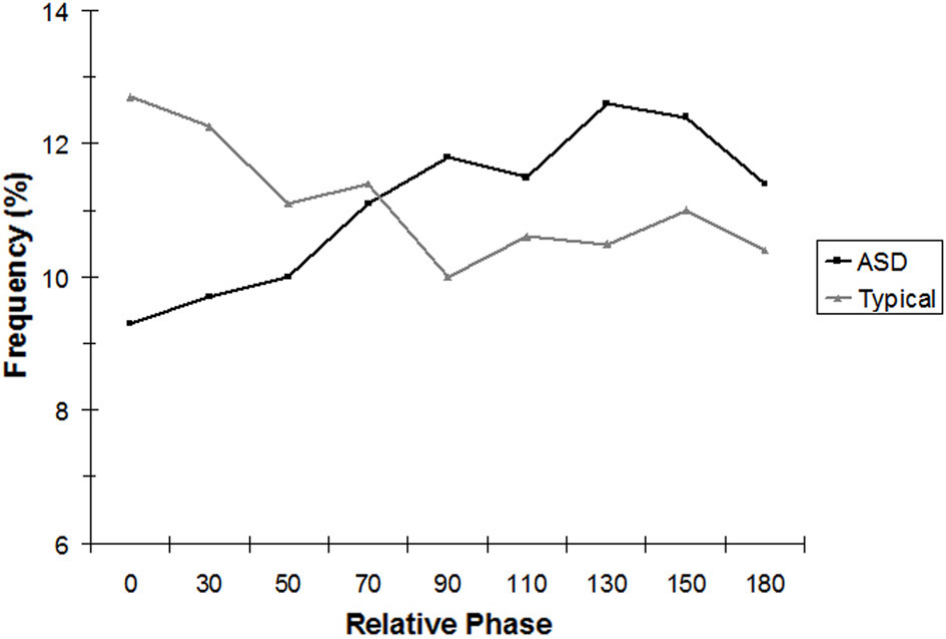
**Analysis of continuous relative phase (CRP), binned into nine equal intervals, for seven ASD and seven typically developing children, age-matched on the visual reception subscale of the Mullen.** For the in-phase (0°) bin only, the effects of group were statistically significant.

Repeating the 2 × 9 mixed ANOVA for the age-equivalent-matched subsample alone revealed similar results. The phase region × group interaction was again significant, [*F*_(8, 96)_ = 3.17, *p* < 0.05]. As Figure 3 indicates, the pattern was the same as with the full sample. TD children showed significantly more in-phase coordination (0°) than children with ASD, *t*_(12)_ = 2.66, *p* < 0.05.

### Exploratory analyses

**Figure 4:**
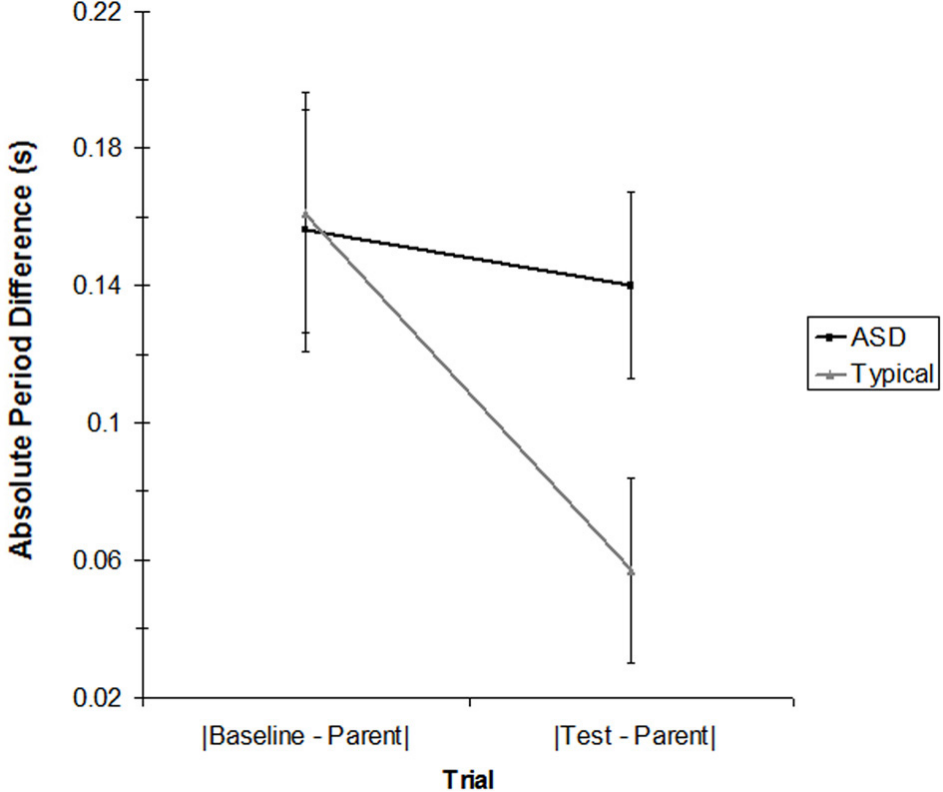
**The absolute value of the children's rocking period for the baseline condition and the test condition compared to the parent's rocking period in the test condition for the ASD and TD groups.** Error bars represent the standard error of the mean.

To explore whether children's rocking period was affected by the parent's rocking period, we compared the child's rocking period on the baseline trial and on the test trial (for the age-matched subsample) to the parent's rocking period on the test trial. Shifts in rocking period toward the parent's period would mean that the child was mimicking the speed of the parent, regardless of whether the child was coordinating the *timing* of their rocking cycle to the parent's. Table 2 presents the average period for each child and parent in the matched sample. As the table indicates, parents were successful rocking at a rate close to their intended period of 1.2 s. Children's baseline rocking periods (when rocking alone) were sometimes longer and sometimes shorter than that of their parents. To determine whether the children's rocking period in the test trial (i.e., when they rocked with the parent) was closer to the parent's period than happened to occur by chance in the baseline trial, the parent's rocking period was subtracted from the child's baseline trial period and the absolute value of each pair was taken (|Baseline—Parent|). This value was compared to the absolute value of the parent's rocking period subtracted from the child's rocking period in the test trial (|Test—Parent|). Table 3 presents these values for each pair. A 2 (Group: Typical vs. ASD) × 2 (Trial: |Baseline—Parent| vs. |Test—Parent|) mixed ANOVA was conducted, with trial as a within-subjects factor. There was no effect of Group, *F* < 1, but there was a significant effect of Trial, [*F*_(1, 12)_ = 11.36, *p* < 0.01]. This effect was moderated by a significant Trial × Group interaction, [*F*_(1, 12)_ = 6.21, *p* < 0.05]. As Figure 4 indicates, although the periods of both groups of children's movements shifted toward their parents' periods during the course of the study, this effect was weaker in children with ASD.

**Table 2:** **Average periods of child and parent rocking for the matched sample of children**.

**ASD**				**TD**			
**Child no.**	**Baseline**	**W/Parent**	**Parent**	**Child no.**	**Baseline**	**W/Parent**	**Parent**
1	1.10	1.13	1.21	1	1.46	1.13	1.22
2	1.27	1.16	1.34	2	1.52	1.38	1.23
3	1.64	1.12	1.34	3	1.04	1.15	1.20
4	1.05	1.05	1.14	4	1.53	1.30	1.33
5	1.57	1.58	1.30	5	1.16	1.26	1.21
6	1.23	1.38	1.30	6	1.33	1.23	1.23
7	1.17	1.29	1.34	7	1.14s	1.21	1.23
*M*	1.29	1.25	1.28	*M*	1.31	1.24	1.24
*SD*	0.23	0.19	0.08	*SD*	0.20	0.08	0.04

Note: Children's average periods when rocking alone and rocking with their parents, as well as the parents' average periods, are presented in seconds.

**Table 3:** **Differences between parent and child rocking periods, for the matched sample**.

**ASD**			**TD**		
**Pair no.**	**|Baseline—Parent|**	**|W/Parent—Parent|**	**Pair no.**	**|Baseline—Parent|**	**|W/Parent—Parent|**
1	0.117	0.082	1	0.238	0.092
2	0.072	0.183	2	0.292	0.153
3	0.303	0.222	3	0.161	0.051
4	0.093	0.087	4	0.197	0.031
5	0.266	0.280	5	0.048	0.048
6	0.069	0.080	6	0.097	0.006
7	0.167	0.047	7	0.091	0.020
*M*	0.156	0.140	*M*	0.161	0.057
*SD*	0.095	0.088	*SD*	0.088	0.051

Note: The absolute values of the difference between each child's average period (rocking alone, and rocking with parent) and that of his or her parent are presented in seconds.

For exploratory purposes, the percentage of time a child spent in symmetrical rocking (in-phase) with their caregiver was correlated with chronological age and intellectual age. Chronological age was not correlated with spontaneous coordination of rocking, *r* = 0.08, *ns*. However, intellectual age, as assessed by Mullen scores, was significantly correlated with in-phase rocking, *r* = 0.54, *p* < 0.05, a pattern that was stable within both groups.

## Discussion

The present study used a rocking chair paradigm to examine the dynamics of uninstructed social coordination of children with ASD and those with no history of developmental disabilities. Not surprisingly, overall interpersonal coordination levels in children were much lower than seen in previous adult studies using rocking chairs (Richardson et al., 2007). Nevertheless, TD children exhibited significantly more in-phase rocking behavior with their parents than did children with ASD matched using the age equivalent on the visual reception subscale of the Mullen. Furthermore, examining the overall period of children's rocking movements against their parents' revealed that TD children shifted their period to that of their parent to a greater degree than did children in the age-matched sample who were diagnosed with ASD. These differences do not appear likely to be a consequence of the parents' rocking tempo being too dissimilar, on average, to the children's tempo—and therefore in dynamical systems terms, were not outside a natural period basin of entrainment. Parents were able to keep their movements close to the instructed frequency, and as a consequence, the period difference between children and parents was less than 4%. Previous research has shown that this period difference is within the basin of entrainment (Lopresti-Goodman et al., 2008) that allows for unintentional interpersonal coordination to emerge. Given that children in both groups had an equal opportunity to unintentionally coordinate with their parents, it is likely that differences between the two groups of children in their perceptual or motoric processes underlies the differences in observed coordination. However, further research is required to be able to rule out the rival possibility that children with ASD merely paid less attention globally to their parent.

With research failing to support key tenets of a theory of mind account of autism (Carpenter et al., 2001; Sebanz et al., 2005), it is a critical time to look how the motoric deficiencies that underlie autism (Bhat et al., 2011; Gowen and Hamilton, 2013; Grossberg and Seidman, 2006; Isenhower et al., 2012) could link to children's inability to engage in joint attention, joint action, and mimicry of others (e.g., Helt et al., 2010; Kinsbourne and Helt, 2011). The results of the current study suggest that at rather fundamental, low-level of motoric behavior that does not depend on intentional, goal-directed action, there are deficiencies in the social grounding of ASD children's movements. Previous research has provided only limited evidence of a link between deficits of synchrony between parent and child; evidence was lacking that synchrony could be due to a unidirectional coupling of the child to the parent (Kinsbourne and Helt, 2011). The current paradigm, examining children's propensity to be pulled into the orbit of their parents' movement patterns during an engaging interpersonal exchange (i.e., reading a book together), provides evidence that children with ASD do not show movement dynamics comparable to what a coupled oscillator account of the coordination of incidental, non-purposive movements would predict. Clues to deficiencies in sociality in ASD may lie in understanding more basic perceptual, attentional (e.g., Liss et al., 2006), and movement abnormalities that often may be the earliest detectable clue that a child has ASD (Grossberg and Seidman, 2006). Marsh et al. (2006) suggest that the ability to time, coordinate, and flexibly adapt our movements with others, may underlie or contribute significantly to our ability to engage others socially. Deficits in intra-personal (within a person) coordination, therefore, may reduce the ability to coordinate interpersonally (between people) and to become moored in a social environment.

Further research is required before such conclusions can be definitively drawn, however. A primary limitation of the current study is its small sample. Future research should replicate and extend these findings, using a wider range of synchrony behaviors across more participants. In the current study, intellectual age was correlated with how much synchrony occurred. Further research would also be needed to rule out differences in the ASD group's degree of overall attention to the adult, or differences in their ability to attend simultaneously to the story and the rocking rate. Recent evidence suggests that for some passive mimicry tasks (e.g., facial movement when viewing a face dynamically expressing emotions), ASD are not impaired in automatic imitation, provided attention is carefully controlled (Press et al., 2010). Whether similar success of ensuring attention could occur for imitation that also requires *temporal* coordination of one's movements with another (as in rocking synchrony) is a critical issue.

Moreover, intervention research is needed to explore the conditions under which interventions will impact interpersonal coordination of movement, and to determine whether motoric-based interventions can have an impact on the sociality deficits of children with ASD. The rationale of this approach is that by focusing the child's attention on the adult's movements, and facilitating simple motoric movement synchrony, individuals can be pulled into the orbit of another, becoming a social unit of perceiving and acting. This is a necessary condition, we suggest, for becoming a fully functional and responsive social actor in more complex interactions with others.

### Conflict of interest statement

The authors declare that the research was conducted in the absence of any commercial or financial relationships that could be construed as a potential conflict of interest.
